# Preparation of graphene oxide liquid crystals with long-range highly-ordered flakes using a coat-hanger die†

**DOI:** 10.1039/d1ra01241j

**Published:** 2021-04-22

**Authors:** Qixin Wu, Hao Guo, Tianxiang Hua, Lilan Zhao, Lingying Li, Bo Qian

**Affiliations:** School of Nano Technology and Nano Bionics, University of Science and Technology of China China; Suzhou Institute of Nano-Tech and Nano-Bionics, Chinese Academy of Sciences China

## Abstract

Graphene oxide (GO) was discovered as a liquid crystalline (LC) phase formation in its water dispersion and expanded to a large number of applications, such as highly ordered GO sheets papers, films, and foams. However, there are still few efficient ways to prepare graphene oxide liquid crystals (GOLCs) with long-range highly ordered flakes. In this work, after carefully studying the rheological properties of GO aqueous dispersions at different concentrations, we have provided a new method to prepare holistically-oriented GOLCs through a designed coat-hanger die. Further, by simulating the extrusion process in the slot of the coat-hanger die, the die's dimensional sizes were optimized to apply efficient shear force on GO dispersions. Then, GOLCs with long-range highly ordered flakes of different GO concentrations were prepared using this method. Finally, a GO foam with a highly ordered structure was prepared using a layer-by-layer method, which exhibited improved conductivity compared to that of normal disordered GO foams after chemical reduction.

## Abstract 2

Graphene oxide liquid crystals with long-range highly ordered GO flakes were successfully prepared by a designed coat-hanger die.

## Graphical abstract

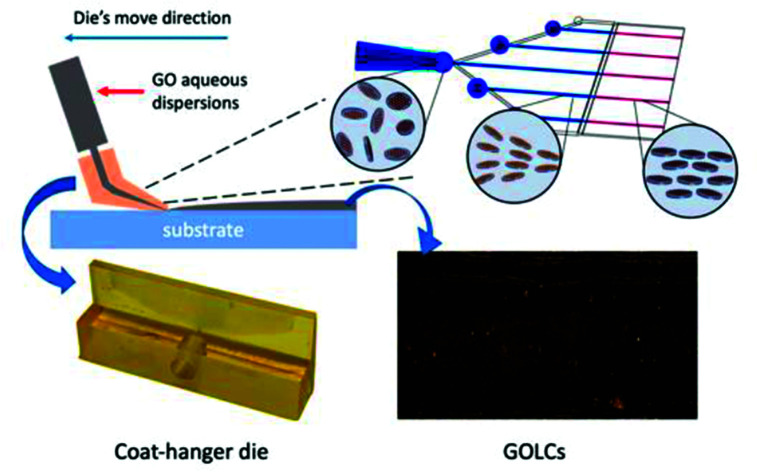

## Introduction

Graphene, the first one-atom-thick material, opened the door of research on two-dimensional (2D) materials in the past decade, and has exhibited versatile applications because of its unique mechanical, electrical and other physical properties.^1,2^ In some large-scale applications of graphene, such as graphene film,^3^ paper^4^ and foam,^5^ graphene oxide (GO) is the main precursor of graphene-based materials due to its oxygenated form of monolayer graphene platelets and can be dispersed in various solvents.^6,7^

GO aqueous dispersion show phase transitions from isotropic phase to liquid crystalline (LC) mesophases depending on their concentrations;^8^ the phase transitions are primarily because of the dimensional anisotropy of GO sheets in the solvent process.^9^ These self-alignment GO sheets in the nematic phase make graphene oxide liquid crystals (GOLCs) a potential multifunctional material due to their local orientation of GO flakes, which exhibit many additional properties such as strong mechanical strength, great flexibility^10^ and conductivity^11^ in reduced GO materials. Since the discovery of the liquid crystalline phase in graphene oxide several years ago, many theories and applications on GOLCs were researched extensively and exhaustively. Those works give GOLCs versatile applications such as high-performance nanocomposites,^12^ optical materials^13,14^ and energy-storage materials.^15^ To date, however, the application of GOLCs as promising materials in various fields has not yet been realized, mainly because of the practical limitations caused by the fabrication of GOLCs with highly ordered GO flakes. The main method to fabricate GOLCs is by immobilizing for several weeks, also known as the self-assembly method.^16^ This method is very time-consuming and cannot fabricate large-sized GOLCs with a holistic orientation of GO flakes, which limited the further applications of GOLCs. Magnetic or electric field can induce the alignment of GOLCs, however, this method is very limited because it induces only a low concentration and small size (<5 μm) of GO flakes, and the orientation of GO sheets will disappear after removing the magnetic or electric field, which is unsustainable.^13,17,18^ Although some researches on the rheological behaviour of GO dispersions in water or various organic solvents for self-assembly of GOLCs have been performed a couple of years ago, there is still no efficient way to fabricate highly aligned GOLCs based on their rheological properties.^16,19^

**Scheme 1:**
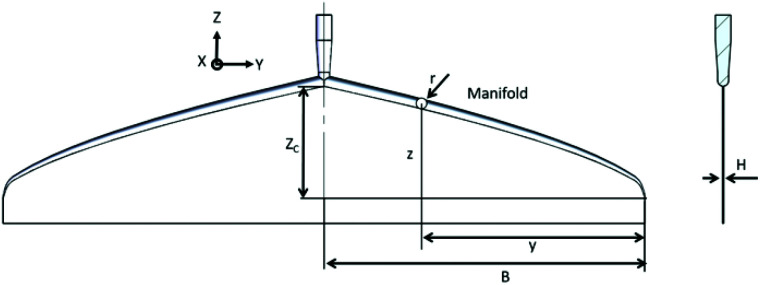
Schematic diagram of coat-hanger die, dimensions and axes *x*, *y* and *z*.

In this work, inspired by the research on rheological properties of GOLCs, we designed a coat-hanger die (Scheme 1) and established a one-step method to produce large-sized GOLCs with long-range highly-ordered GO flakes. The slot size of the coat-hanger die was carefully investigated based on Matsubara's mathematical model^20^ and the specific die sizes were accurately calculated according to the rheological parameters of GO dispersions, such as shear rate to shear viscosity and shear modulus relations. Then, we used finite element analysis to simulate the GO flow in the designed coat-hanger die for verifying the rationality of the die's channel. A photo-curing three-dimensional (3D) printer with 10 micron resolution was used to fabricate the coat-hanger die, the outlet width was demonstrated to be only 100 μm. Such a narrow outlet can generate ultra-high shear force to GO aqueous dispersions and produce long-range orientational GO flakes. Moreover, it is possible to fabricate ultra-large and highly aligned GOLCs on the substrate in a very short time (several seconds). Finally, we prepared a highly ordered structure GO foam by this method, the reduced GO foam exhibits improved conductivity compared to normal disordered GO foams.

## Results and discussion

To study the specific parameters of the coat-hanger die, it is necessary to investigate the rheological behaviour of GO aqueous dispersions. In this study, five different concentrations (2 mg mL^−1^, 4 mg mL^−1^, 6 mg mL^−1^, 8 mg mL^−1^ and 10 mg mL^−1^ marked as GO-2, GO-4, GO-6, GO-8 and GO-10, respectively) of well-dispersed GO aqueous dispersions were used as research samples, and the mean size of GO nanosheets was about 30 μm (Fig. S1, ESI†), which was confirmed by scanning electron microscope (SEM) measurements.^21^

**Fig. 1:**
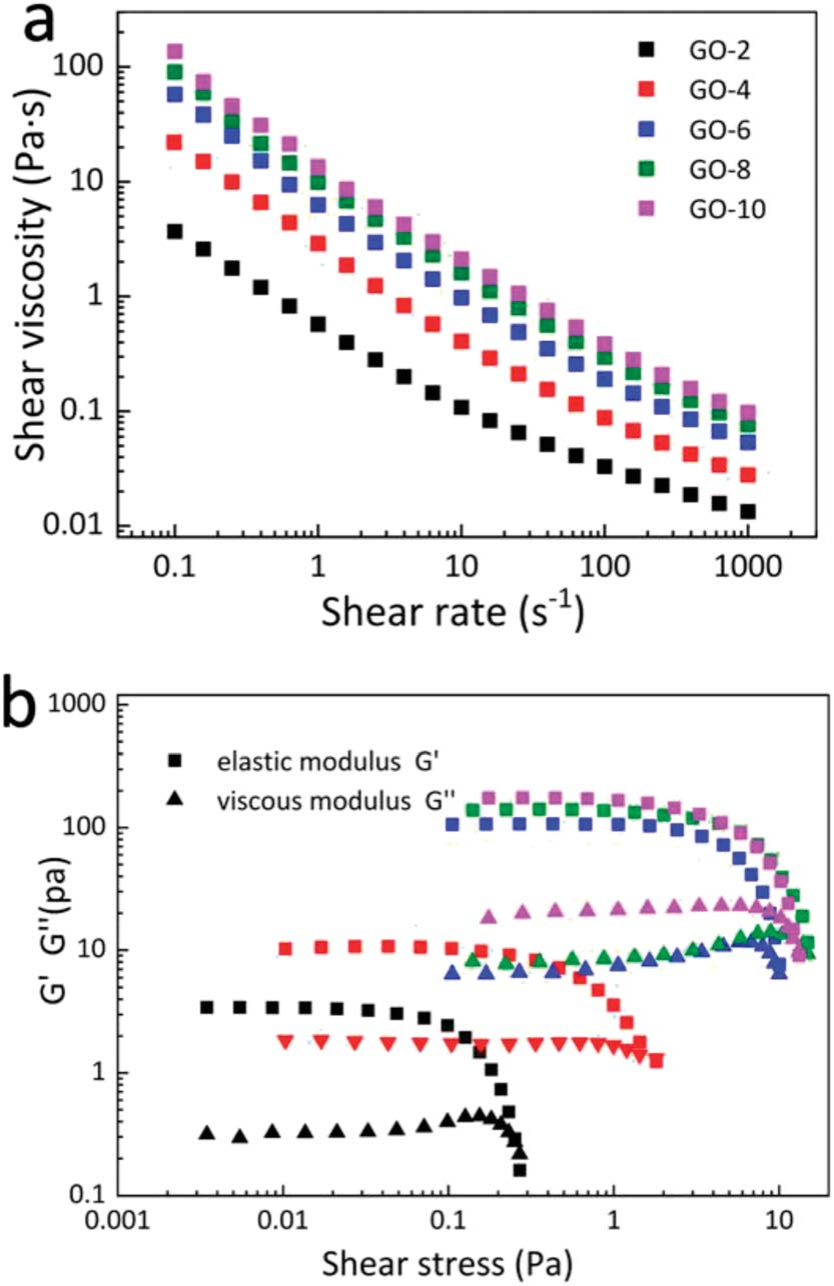
Rheological behaviour of GO aqueous dispersions. (a) The flow curves show viscosity rapidly increases as GO dispersions concentrations raises from 2 mg mL^−1^ to 10 mg mL^−1^, and viscosity of all 5 different GO dispersion concentrations decreases as shear rate increases. (b) Different elastic modulus (*G*′) and viscous modulus (*G*′′) of 5 GO dispersions.

Fig. 1a shows the logarithmic plot of the dependence of shear viscosity on the shear rate of the GO dispersions with different concentrations measured using a rheometer (see the Experimental section). Generally, for the shear rate from 0.1 s^−1^ to 1000 s^−1^, the viscosities of all 5 GO dispersions decrease exponentially with almost the same trend and parallel to each other except for a small deviation in the curve of GO-2 on the small shear rate side. The curve value increases with an increase in the concentration. For the lowest curve of GO-2, the viscosity changes from 4 Pa s to 0.015 Pa s. For the highest curve of GO-10, the viscosity changes from 110 Pa s to 0.15 Pa s. This indicates a shear-thinning property and proves that GO water dispersion is a typical non-Newtonian fluid^22,23^ for all 5 GO samples. This is a typical lyotropic LC behaviour and was caused by the highly ordered structures along the shear direction.^24^ Note that as the GO concentration increases, the curves of the samples become closer to each other, mainly because GO sheets are oriented more parallel to each other to minimize the excluded volume, which contributes to less interaction between GO sheets.^16^ Moreover, the elastic *G*′ (storage) moduli are larger than viscous *G*′′ (loss) moduli in 5 concentrations of GO dispersions as shown in Fig. 1b. The dominance of *G*′ along with *G*′′ suggests a gel-like behaviour of GO dispersions and can form single-phase nematic liquid crystals by self-assembly method, which means that the nematic liquid crystal phase will sustain well after applying shear stress to GO dispersions. This kind of rheological behaviour of GO provides a possibility for fabricating GOLCs *via* established fabrication techniques that normally process gel-like materials.^19^

The coat-hanger dies are widely used in non-Newtonian fluid fabrication process for the sheets and films.^25^Scheme 1 shows the schematic diagram of the coat-hanger die with archly tapered manifolds, where *B* and *H* represent half of the coat hanger width and slot gap, respectively.^26^ The power-law model was used to describe the relationship between the shear stress (*τ*) and shear rate (*γ*):

1 *τ* = *Kγ*^*n*−1^,

where *K* and *n* refer to the non-Newtonian viscosity in a standard state and the flow behaviour index, respectively.^23^ Those two parameters were calculated based on the rheological parameters that were investigated, as shown in Fig. 1, and are crucial to determining the die's dimensions shown in Scheme 1, the specific calculation process is in the ESI.†^25^ Then, we calculated 5 groups of *K* and *n* based on 5 different concentrations of GO and get 5 different specific values of die's manifold size (Eqn (S1), ESI†). Table 1 shows the values of *K* and *n* for different concentrations of GO dispersions in this work.

**Table 1:** Power-law parameters (*K* and *n*) of GO dispersions

GO dispersions	*K* (kg cm^−2^ s)	*n*
GO-2	0.81	0.385
GO-4	3.80	0.260
GO-6	9.28	0.237
GO-8	14.14	0.235
GO-10	19.74	0.225

Then, 3D models of 5 different coat-hanger dies were drawn for further fabrication and simulations according to different *K* and *n*. To ensure that the coat-hanger die can induce uniform shear stress in the GO aqueous dispersions, simulating the extrusion process of GOLCs in the die's slot is very necessary.^27^

**Fig. 2:**
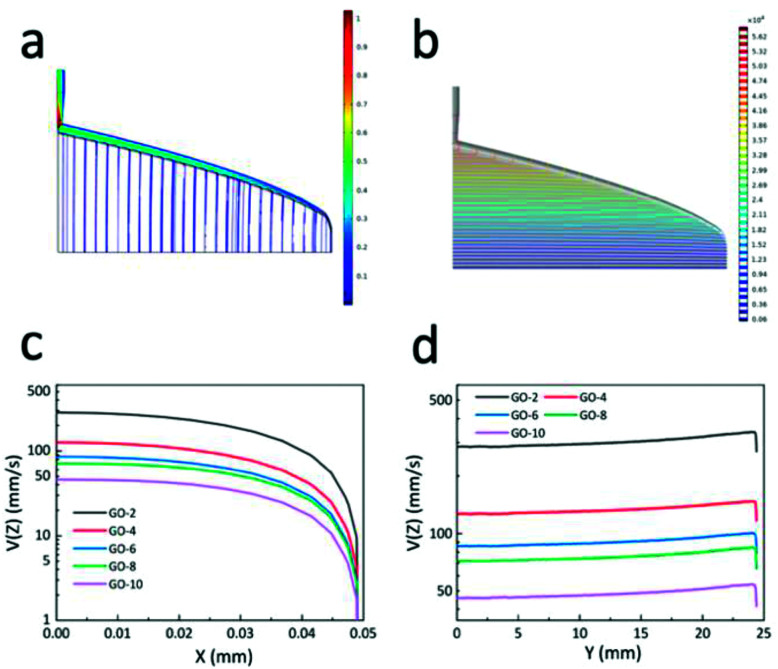
(a) The distribution of flow velocity in die's right slot and manifold. (b) The distribution of pressure in die's right slot and manifold. The flow velocity of 5 different GO dispersions in *x*-axis (c) and in *y*-axis (d) at the outlet of the die.

Fig. 2 illustrates the fluid simulation results of the extrusion process. Note that the coordinates in Fig. 2 strictly follow the definition in Scheme 1. For the sake of illustration, the die, designed based on GO-6, was chosen here to elaborate the simulation progress as shown in Fig. 2a and b. The fluid parameters were determined from the rheology of GO dispersions, the pressure applied to the GO dispersions was 50 kPa. Fig. 2a shows simulation results of the flow velocity in the die's right slot (the coat-hanger die has a symmetrical structure; the left part of the die is the same as the right part), and there is a uniform distribution of contours velocity magnitude in the slot. This means that GO dispersion flow was extruded from the die's outlet at the same velocity in the *y*-axis direction, which is confirmed in Fig. 2d. Fig. 2b shows the isobar in the die's slot, it is clear that the pressure at the entrance of the slot was much greater than that at the end. This indicates that GO dispersion flow along the manifold direction once they were extruded from the entrance of the die into the manifold section. Furthermore, as shown in Fig. 2b, the pressure was gradually decreased as the GO dispersions flowed down in the die's slot since the manifold was the region to transport GO dispersions and the slot was the main region that induces uniform shear stress on the GO dispersions.^28^ Then, the velocity in the slot of the other 4 different GO concentrations was simulated based on each designed coat-hanger die. Fig. 2c and d show the velocity of 5 different GO dispersions on the *x*-axis and *y*-axis, respectively. As we know from the rheological properties in Fig. 1, the viscosity increases with increasing GO concentration, and at the same time, the flow velocity in the slot decreases at the same pressure (50 kPa), as shown in Fig. 2c. At the centre of the slot, the flow velocities range from 4.6 cm s^−1^ for GO-10 to 28.5 cm s^−1^ for GO-2. Fig. 2d shows the flow velocity at the outlet of the die in the *y*-axis, which showed a uniform distribution of GO dispersion when extruding onto the substrate. For extruding a constant thickness of GOLCs, such uniform velocity distribution inside the die is desirable. Moreover, these different velocities give an important reference for the movement speed of the die in the fabrication progress of highly ordered GOLCs (see ESI†). Other additional simulation results such as shear rate distribution and shear viscosity were in Fig. S2.† It is worth mentioning that the size of the slot gap has an important influence on the shear process, however, limited by the preparation process of the coat-hanger die, the slot gap size was set as a controlled variable here. We also designed 3 dies with different slot gap sizes and simulated the effect of the different gap sizes on the shear rate in Fig. S3.†

**Fig. 3:**
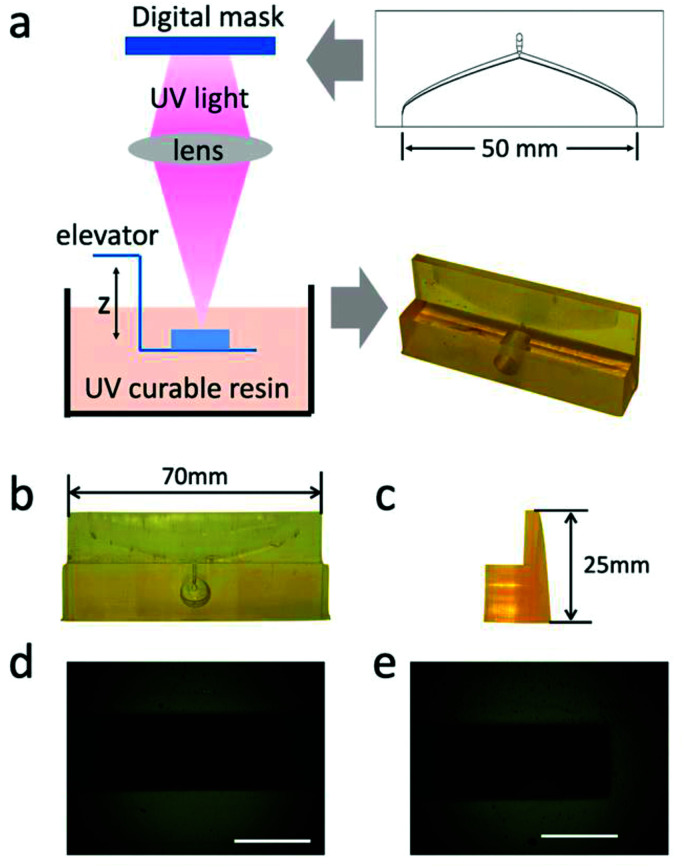
(a) Schematic diagram of the fabrication process of the coat-hanger die by photo-curing 3D printer, the outlet length is 50 mm. (b) and (c) are front view and side view of coat-hanger die, respectively. The length and height are 70 mm and 25 mm. (d) and (e) are the optical microscope photos of the center and edge of the die's outlet. Scale bar represents 100 μm.

To verify the simulation results, it is necessary to prepare the coat hanger to die with high precision. A photo-curing 3D printer, which uses micron-level patterned UV light to cure the photosensitive resin was used to prepare 5 different coat-hanger dies^29,30^ for the different concentrations of GO dispersions with only slightly modified dimensions of the dies (see ESI†). The preparation progress of the coat-hanger die is shown in Fig. 3a. From the front and side views as shown in Fig. 3b and c, it can be observed that the printed coat-hanger die has a smooth surface, which is important for fluid flow. The measured length and height of the die were 70 and 25 mm, which were exactly the design dimensions. Fig. 3d and e are the optical microscope images of the middle and edge of the die's outlet. The outlet size was about 100 um in width and 50 mm in length, which is consistent with the design dimensions.

**Scheme 2:**
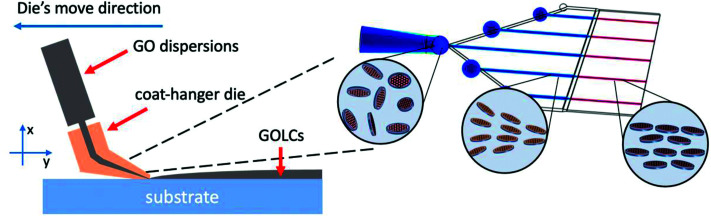
Schematic illustration of fabrication process and the state of GO sheets change from isotropic to nematic phase in the die's channel.

**Fig. 4:**
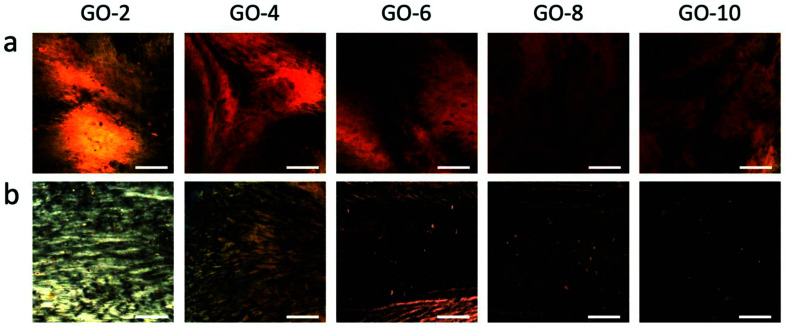
Polarized optical microscope (POM) images of (a) GO dispersions prepared by drop dispensing and (b) GO dispersions prepared by extrusion from the coat-hanger die. Scale bar represents 200 μm.

Scheme 2 shows the fabrication process of GOLCs with the designed coat-hanger die, which is connected to a modified gel extruder. The inset diagram on the right is the schematic illustration of GO flakes changing from isotropic to nematic phase in the die's slot. After adjusting the angle of the die's slot and substrate (the die's slot should be kept as parallel as possible to the substrate), the GO aqueous dispersions were extruded from the die onto the transparent SiO_2_ substrate under precisely controlled air pressure (50 kPa). To ensure that the GO dispersion was relatively static to the substrate in the *y*-axis direction, the moving speed (8.6 cm s^−1^ for GO-6) of the die was carefully controlled and determined by the flow velocity at the centre of the die's outlet, which was obtained from the simulated flow velocity in Fig. 2d. Then, a polarized optical microscope (POM) was used to observe the birefringence of GOLCs after it was extruded onto a transparent SiO_2_ substrate. Fig. 4 shows a comparison of POM microscopic images of GO aqueous dispersions prepared using the drop dispensing process (Fig. 4a) and the extruding process by the die (Fig. 4b). It is easy to distinguish that the birefringent optical texture related to the nematic phase at different concentrations of GO dispersions in Fig. 4a, which reflects the local orientation of the GO sheets originating from the self-assembly process after drop dispensing.^8^ Compared to Fig. 4a and b shows the long-range ordered birefringent optical texture of extruded GOLCs for GO-2 and GO-4, which becomes darker and less birefringent texture part as the concentration of GO dispersions increases. GO-10 exhibits a dark phase close to zero birefringence. This is mainly due to the highly aligned GO nanosheets, which are parallel to the substrate, induce light propagation parallel to the optic axis and is known as the pseudo-isotropic orientation, like the lack of birefringence observed in the isotropic phase.^9^ As shown in Fig. 1b, the dominance of *G*′ (storage) will keep the highly aligned GO flakes to sustain well after the extrusion progress. Some birefringent textures appeared at the edge of the extruded GO dispersions, as shown in Fig. S4,† which is mainly because the surface tension of the GO dispersions changes the highly ordered GO flakes from a holistic orientation to a local orientation.

**Fig. 5:**
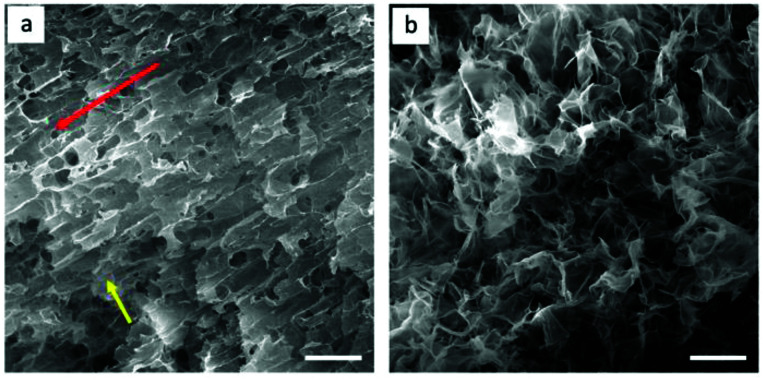
SEM images of GO foams. (a) GO foam prepared by the coat-hanger die, red arrow represents the orientation of GO flakes, yellow arrow points out the connection between different layers. (b) Disordered GO foams prepared by drop dispensing process. Scale bar represents 200 μm.

GO-10 foams with highly aligned GO flakes were prepared by repeating the extruding process layer by layer 50 times after freeze-drying. For comparison, GO-10 foams of similar sizes were prepared by drop-dispensing process. Fig. 5 shows the structural details on the alignment of GO foams prepared by the extrusion process (Fig. 5a) and drop dispensing process (Fig. 5b). In Fig. 5a, GO flakes were aligned in the same direction after extrusion progress, as shown by the red arrow, and exhibits anisotropic structure–property. It is worth noting that GO flakes bonded well between layers, as shown by the yellow arrow, which is mainly due to the strong self-assembly force of van der Waals force, π–π stacking and hydrogen bonding.^18^ In comparison, Fig. 5b shows normal GO foams with no clear orientation of GO flakes, which were not extruded from the coat-hanger die. To investigate the conductivity of the highly aligned GO structure, we reduced these two GO foams by the chemical reduction method to obtain reduced GO (rGO) foams. The Raman spectra of GO and rGO foam are shown in Fig. S5.^31^† The conductivity of highly aligned rGO foam reached 92 S m^−1^, which was larger than that of rGO foam without alignment of the rGO flakes (32 S m^−1^). Those highly ordered rGO flakes can provide higher carrier mobility compared to that of disordered rGO flakes.

## Experimental

The initial GO dispersion was 1.14 wt% (purchased from Timesnano Inc, Chengdu) and has a mean lateral size of about 30 μm. Then, pure GO nanosheets were obtained *via* a vacuum filtration method and were dissolved in deionized water by slight ultrasound treatment to get four different concentrations of GO aqueous dispersions: 2 mg mL^−1^, 4 mg mL^−1^, 8 mg mL^−1^ and 10 mg mL^−1^, for convenience, the different concentrations of GO dispersions were marked as GO-2, GO-4, GO-6, GO-8 and GO-10, respectively.

Rheometer (Kinexus Lab+, NETZSCH Instruments) was used to investigate the rheological properties of GO dispersions with a conical-shaped spindle (angle: 2°, diameter: 40 mm). About 1 mL of GO dispersions were loaded carefully onto the sample stage and taken not to touch the conical spindle after several calibrations of the rheometer. Shear stress and viscosity were measured at shear rates between 0.1 and 1000 using logarithmic steps for two complete (ascending and descending) cycles. After reloading the sample, the complex shear modulus (elastic and viscous component) was measured at complex shear stress between 1 and 100 using logarithmic steps. After calculating the specific parameters of the coat-hanger die's channel according to its rheological properties, a 3D model was drawn for 3D printing and simulations. The 3D finite element method was adopted to solve the three-dimensional GO dispersion flow in the coat-hanger die. A photo-curing 3D printer (NanoArch S140, BMF Material Technology Inc.) was used to fabricate the coat-hanger die and OPG microscope to verify scales of the die's outlet. A modified gel-extruder (Axxon VB-200) was used to provide stable air pressure and movement of the coat-hanger die. A polarized optical microscope (POM) was used to investigate the birefringence of GO dispersions after extrusion progress.

GO foams were fabricated by freeze-drying method after extruding onto a glass substrate. The scanning electron microscope, SEM (Quanta FEG 250, EFI, USA), was used to observe the specific alignment of GO sheets. Hydroiodic acid/acetic acid in the ratio of 1 : 2 at the temperature of 40 °C for 24 hours was used to reduce GO foams and get rGO foams. Chemical compositions of GO and rGO foams were determined by Raman microscopy (LABRAM HR, Horiba-JY, Japan). Electrical conductivity was determined by a 4-probe resistivity meter (ST-2258A, Suzhou JingGe Technology Inc.).

## Conclusions

In summary, the rheological properties of GO aqueous dispersions were investigated for the design of the coat-hanger die. The simulation results showed the progression and principles of GO flakes change from the isotropic phase to the nematic phase in the die's slot. GOLCs with long-range highly ordered GO flakes with a width of 50 mm and length of 70 mm were successfully fabricated in the designed coat-hanger die, and anisotropic GO foams were prepared by freeze-drying the extruded GOLCs, which exhibited excellent electrical conductivity compared with normal GO foams after chemical reduction. We think that this study may provide an efficient way to prepare GOLCs with highly aligned GO flakes, which may improve the mechanical and electrical properties of GOLCs-based materials and expand the application range of GOLCs, such as in the GO film, paper and foams.

## Conflicts of interest

There are no conflicts to declare.

## Supplementary Material

RA-011-D1RA01241J-s001
