# Supplementary material for: Preparation of graphene oxide liquid crystals with long-range highly-ordered flakes using a coat-hanger die
Source: RSC Adv. 2021 Apr 22;11(25):15085–90. doi: 10.1039/d1ra01241j (PMC8698725; doi:10.1039/d1ra01241j)
Supplement: RA-011-D1RA01241J-s001 [file RA-011-D1RA01241J-s001.pdf]

Electronic Supplementary Material (ESI) for RSC Advances.
This journal is © The Royal Society of Chemistry 2021

Electronic Supplementary Information (ESI) for RSC Advances.

Supporting Information

Preparation of Graphene Oxide Liquid Crystals with Long-range Ordered Flakes through the Coat-hanger die

Qixin Wu, Hao Guo, Tianxiang Hua, Lilan Zhao, Lingying Li and Bo Qian*
School of Nano Technology and Nano Bionics, University of Science and Technology of China. E-mail: bqian2010@sinano.ac.cn

Table of Contents

1. **Mean lateral size of GO sheets**
2. **Mathematical formulation of the coat-hanger die**
3. **Simulation results of shear rate and shear viscosity**
4. **Birefringent texture of GOLCs at the edge**
5. **Raman spectra of GO and rGO foams**

Lateral mean size of GO flakes

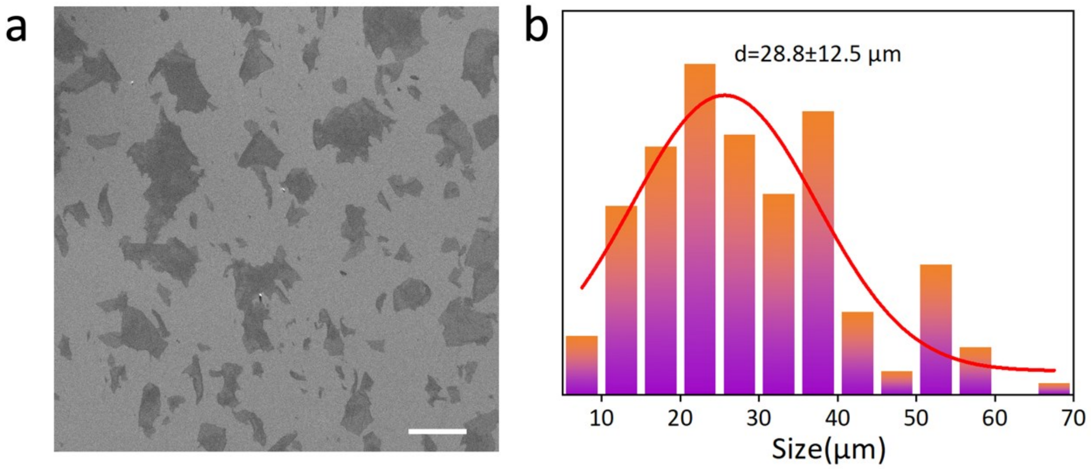

Fig. S1 (a) SEM of GO sheets of GO dispersions and (b) histogram of the sizes of GO sheets. The mean lateral size of GO flakes are around 30um.

Mathematical formulation of the coat-hanger die[1]

The coat-hanger die in scheme 1 is shown here with following assumptions:

1) Polymer melt viscosity can be described by so called power-law equation (Eq 1).
2) Melt temperature is uniform throughout the flow stream.
3) The manifold is a circular tube and the coat-hanger has a constant gap.
4) Melt flow is laminar.
5) Melt in the coat-hanger section flows only along the machine direction.
6) Melt in the manifold flows only in the z-direction.
7) Residence time are the same in the die's manifold and slot.

According to the power law equation in Eq 1
The manifold radius $R(y)$ in y-axis direction expressed as follows:

$$R(y) = m^{\frac{1}{3(n+1)}} \cdot \pi^{-\frac{1}{3}} \cdot \left\{\frac{(3n+1)}{2(2n+1)}\right\}^{\frac{n}{3(n+1)}} \cdot H^{\frac{2}{3}} \cdot (B-y)^{\frac{1}{3}} \qquad (S1)$$

Where m is the ratio of residence time in the manifold to residence time in the slot; n is the flow behavior index in the Eq 1; H is the gap of the coat-hanger die section; B is the half of the coat-hanger width.
In our designed coat-hanger die, $m = 1$ according to the assumptions, H sets as 100 µm, B sets as 25 mm.
It is worth to mention that H is a control variable in our design. Theoretically, the shear rate is

inversely proportional to the gap size of the coat-hanger die according to Eq S2, which means that a smaller size of slot gap can better extrude the highly ordered GOLCs. The reason of H sets as 100 μm was that 100 μm was the minimum size that our 3D printer can stably print.

$$\dot{\gamma}_c = \pm\left(\frac{2n+1}{n}\right)\left(\frac{2Q_0}{BH^2}\right) \qquad (S2)$$

Where Q_0 is the half of the total volumetric flow.

The height of manifold to the slot outlet of the

$$z(y) = \int_0^z dz = \int_0^y \left\{\frac{k'}{(B-y)^{\frac{2}{3}} - k'}\right\}^{\frac{1}{2}} dy$$

$$= -\frac{3}{2}\cdot k'^{\frac{1}{2}}\left[(B-y)^{\frac{1}{3}}\cdot\sqrt{(B-y)^{\frac{2}{3}} - k'} + k'\ln\left\{(B-y)^{\frac{1}{3}} + \sqrt{(B-y)^{\frac{2}{3}} - k'}\right\}\right]^y \qquad (S3)$$

Where k' :

$$k' = m^{\frac{-2(3n+1)}{3(n+1)}}(\pi H)^{\frac{2}{3}}\left\{\frac{(3n+1)}{2(2n+1)}\right\}^{\frac{4n}{3(n+1)}}$$

The center height of the coat-hanger die, Z_c, is calculated as:

$$Z_c = \int_0^{L-k^2} \left\{\frac{k'}{(B-y)^{2/3} - k'}\right\}^{\frac{1}{2}} dy$$

$$= -\frac{3}{2}\cdot k^{\frac{1}{2}}\left[k'\log_e k^{\frac{1}{2}} - B^{\frac{1}{3}}\sqrt{B^{2/3} - k'} - k'\ln\left\{B^{\frac{1}{3}} + \sqrt{B^{2/3} - k'}\right\}\right] \qquad (S4)$$

Then, we calculated the dimensions of the coat-hanger die:

GO dispersions	n	K	B (mm)	H (μm)	R_0 (mm)	Z_c(mm)
GO-2	0.385	0.81	50	100	0.1553	3.9977
GO-4	0.260	3.80	50	100	0.1567	4.0777
GO-6	0.237	9.28	50	100	0.1570	4.0959
GO-8	0.235	14.14	50	100	0.1571	4.0976

GO-10	0.225	19.74	50	100	0.1572	4.1060

Simulation results of shear rate and shear viscosity

The shear rate and shear viscosity of the flow at the die's outlet in x-axis. The extrusion pressure was set as a controlled variable here.

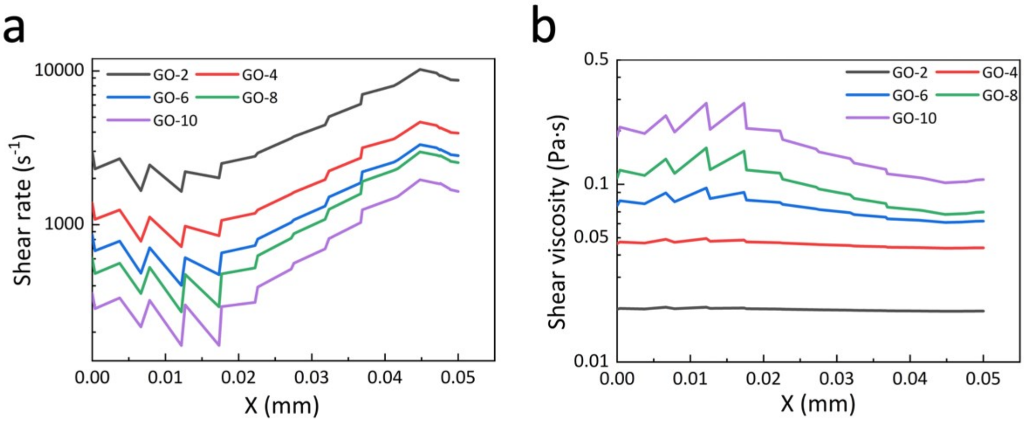

Fig. S2 Shear rate (a) and shear viscosity (b) of the simulation of the coat-hanger die in x-axis.

To investigate the changes in shear rate at different slot gap sizes, we designed three different coat-hanger die with 100 μm, 150 μm and 200 μm gap sizes according to the rheological properties of GO-10. The volumetric flow rate was set as a controlled variable in Fig. S3a to obtain the same velocity of the flow at the center of die's outlet. The shear rate in Fig. S3b was obtained from the differentiation of the flow velocities in Fig. S3a.

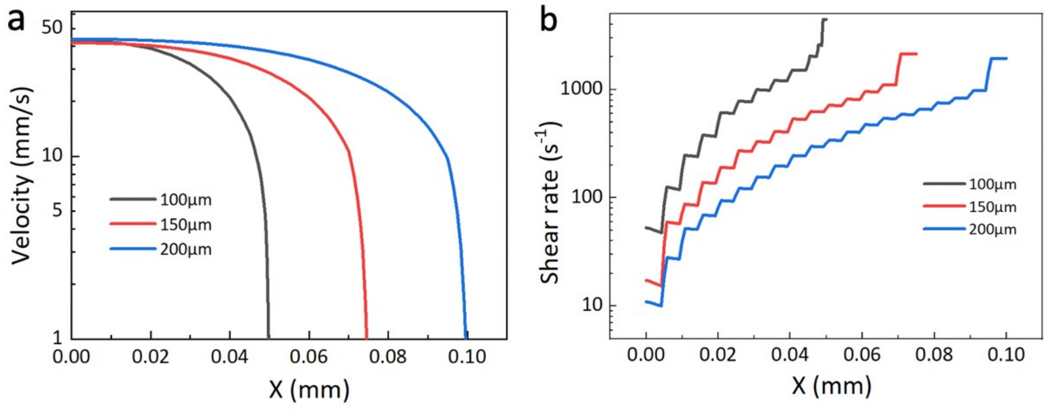

Fig. S3 a) The velocities of the flow at the outlet of the die in x-axis; b) The influence on shear rate of different slot gap sizes of the die; As mentioned that smaller gap sizes will increase shear rate in the die's slot.

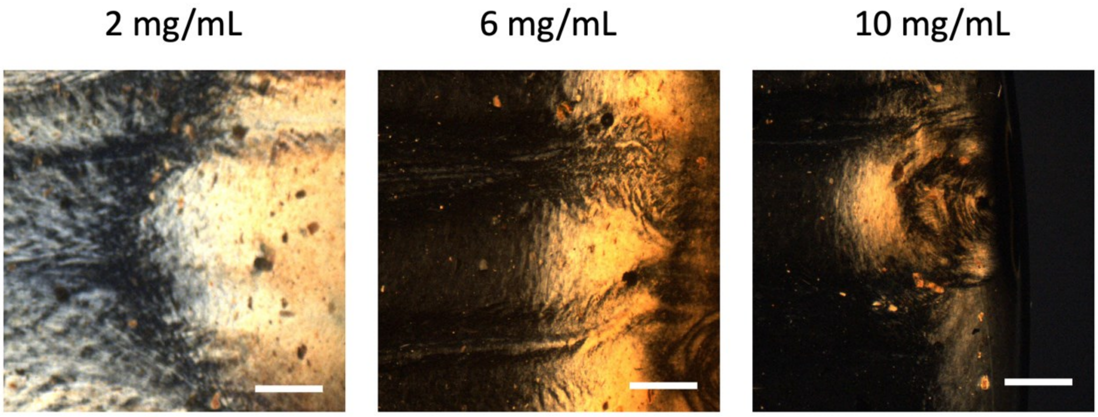

Birefringent texture of GOLCs at the edge

Fig. S4 POM images of GOLCs at the edge of the extruded GO dispersions.

Raman spectra of GO and rGO foams

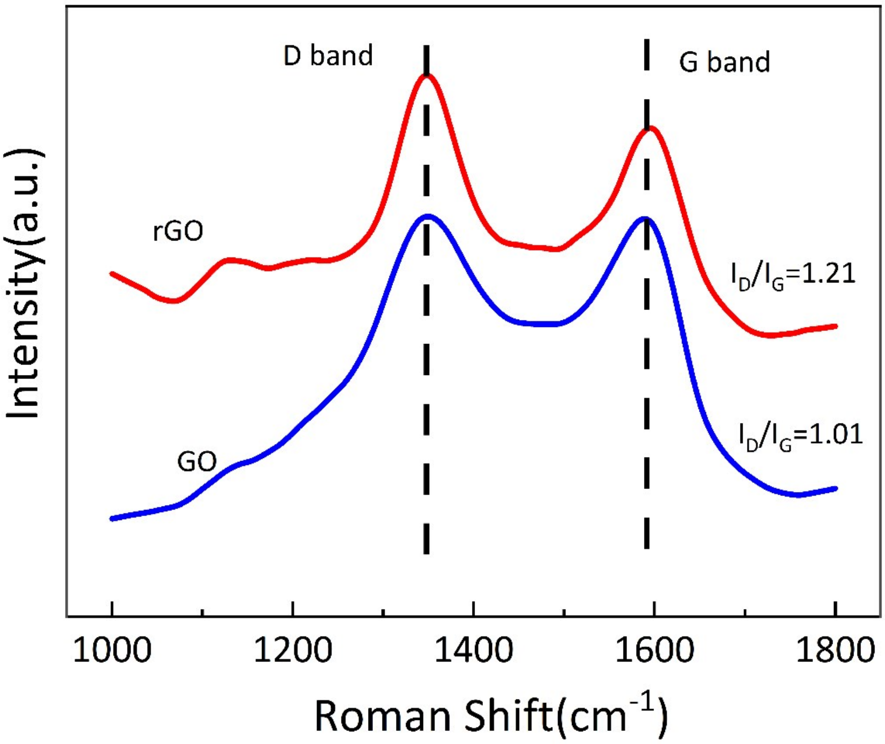

Fig. S5 Raman spectra of GO and rGO foams. I_D/I_G changes from 1.01 to 1.21 for GO and rGO respectively, indicating that the reduction process altered the structure of G-O.

1. Y. Matsubara, *Polym. Eng. Sci.*, 1980, **20**, 716–719.
